# Management of a pregnant woman with Marfan syndrome and aortic root and aberrant right subclavian artery aneurysm: a case report

**DOI:** 10.1093/ehjcr/ytae411

**Published:** 2024-08-09

**Authors:** Inga Voges, Ulrike Hoffmann, Tim Attman, Anselm Uebing

**Affiliations:** Department of Congenital Heart Disease and Paediatric Cardiology, University Hospital Schleswig-Holstein, Arnold-Heller-Str. 3, Campus Kiel, 24105 Kiel, Germany; Department of Congenital Heart Disease and Paediatric Cardiology, University Hospital Schleswig-Holstein, Arnold-Heller-Str. 3, Campus Kiel, 24105 Kiel, Germany; Department of Cardiovascular Surgery, University Hospital Schleswig-Holstein, Kiel, Germany; Department of Congenital Heart Disease and Paediatric Cardiology, University Hospital Schleswig-Holstein, Arnold-Heller-Str. 3, Campus Kiel, 24105 Kiel, Germany

**Keywords:** Marfan syndrome, Pregnancy, Aortic aneurysm, Valve-sparing aortic root replacement, Case report

## Abstract

**Background:**

Marfan syndrome is a genetic connective tissue disorder that commonly affects the cardiovascular, skeletal, and ocular system. The increased risk of developing thoracic aortic aneurysms that can lead to aortic dissection and rupture is the main source of mortality in these patients. Pregnancy-induced changes can further increase the risk for aortic complications, especially in patients with an aortic root diameter > 45 mm.

**Case summary:**

The case of a 26-year-old female with Marfan syndrome who was lost to follow-up for five years and presented to our department being pregnant at 21 weeks is presented. Echocardiography and cardiovascular magnetic resonance (CMR) showed an aortic root diameter of 55 mm and a large aneurysm of an aberrant right subclavian artery. Following multidisciplinary team discussion, valve-sparing aortic root and ascending aortic replacement was performed at 22 weeks of gestation without any complications. During the remaining pregnancy, the patient had frequent clinical and CMR follow-up investigations showing a mild increased size of the subclavian aneurysm. Uncomplicated caesarean delivery was performed at 35 weeks of gestation, and the subclavian artery aneurysm was successfully treated by interventional embolization.

**Discussion:**

Although cardiovascular surgery in our patient during pregnancy was uncomplicated, the case illustrates that pre-pregnancy counselling in Marfan patients is recommended to reduce the risk for mother and child.

**Learning points**

- Pre-conceptional patient counselling is advised in patients with connective tissue disorders.
- Pregnancy in Marfan syndrome carries a significant risk for aortic dilatation and dissection.

## Introduction

Marfan syndrome is an autosomal dominant connective tissue disorder that involves the vascular tree and can lead to aortic aneurysms with the risk of dissection and rupture. Pregnancy significantly increases the risk for aortic dissection, both type A and type B, and seems to be more common in the post-partum period.^1,2^

Current guidelines describe an increased risk for type A dissection during pregnancy in patients with an aortic root diameter > 40 mm.^3,4^ Furthermore, patients with an ascending aortic or aortic root diameter > 45 mm should undergo prophylactic aortic surgery before pregnancy and should be advised against pregnancy.^3,4^

## Summary figure

**Table:** 

Age 12 years	Diagnosis of Marfan syndrome
Age 12–21 years	Regular cardiac follow-up in the congenital heart disease department at a tertiary centre
Age 21–26 years	Lost to follow-up
November 2021, age 26 years	Patient presented to the congenital heart disease department being pregnant at 21 weeks Echocardiography and cardiovascular magnetic resonance (CMR) showed aortic root enlargement (55 mm) and an aberrant right subclavian aneurysm
November 2021, age 26 years, 22 weeks of gestation	Valve-sparing aortic root and ascending aortic replacement was performed after multidisciplinary team discussion
November 2021–February 2022	Biweekly CMR follow-up to assess the size of the right subclavian artery aneurysm
February 2022	Caesarean section at Week 35 of gestation
April 2022	Computed tomography of the aorta
July 2022	Successful interventional embolization of the subclavian artery aneurysm

## Case presentation

We report about a 26-year-old female with Marfan syndrome who was without regular cardiac care for 4 years. Her last available echocardiogram and cardiovascular magnetic resonance (CMR) scan showed aortic root dilatation with a maximum diameter of 42–43 mm. She was also known to have an aberrant right subclavian artery. The implications of pregnancy with her condition were never discussed with her.

The patient presented to the Department of Congenital and Paediatric Cardiology again when she was 21 weeks into her first pregnancy and referred for a foetal echocardiogram.

**Figure 1:**
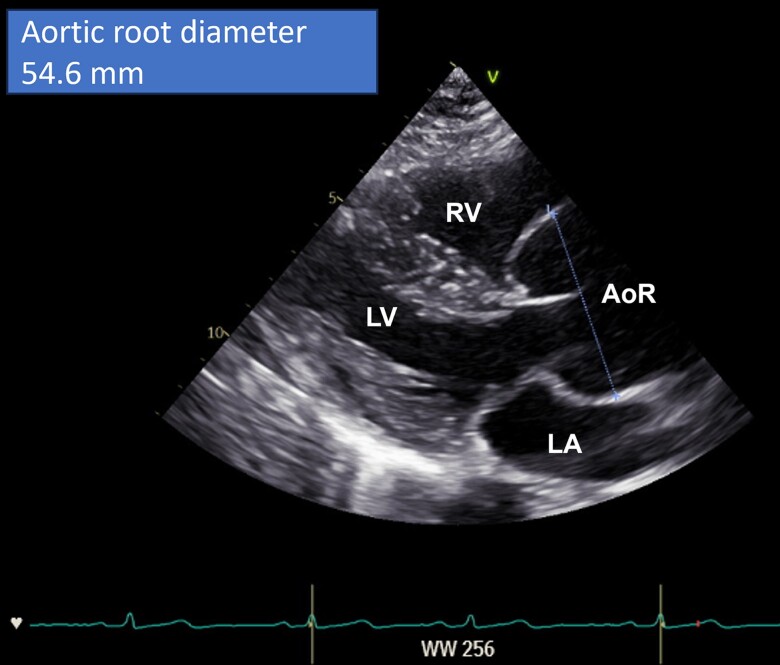
Parasternal long axis view showing the enlarged aortic root (AoR) with a maximum diameter of 55 mm. AoR, aortic root; LA, left atrium; LV, left ventricle; RV, right ventricle.

At the time of presentation, she was clinically well and asymptomatic. She was treated with beta-blockers (metoprolol 47.5 mg twice a day), and this was continued throughout the pregnancy. Echocardiography showed an increased aortic root size compared to her last examination with a maximum diameter of 55 mm (Z-score +6.6, *Figure 1*, *Movie clips 1* and *2*).^5^ There was mild aortic valve and trivial mitral valve regurgitation. Mitral valve prolapse was not observed, and left ventricular function was normal (shortening fraction was 32%, and left ventricular ejection fraction was 59%).

**Figure 2:**
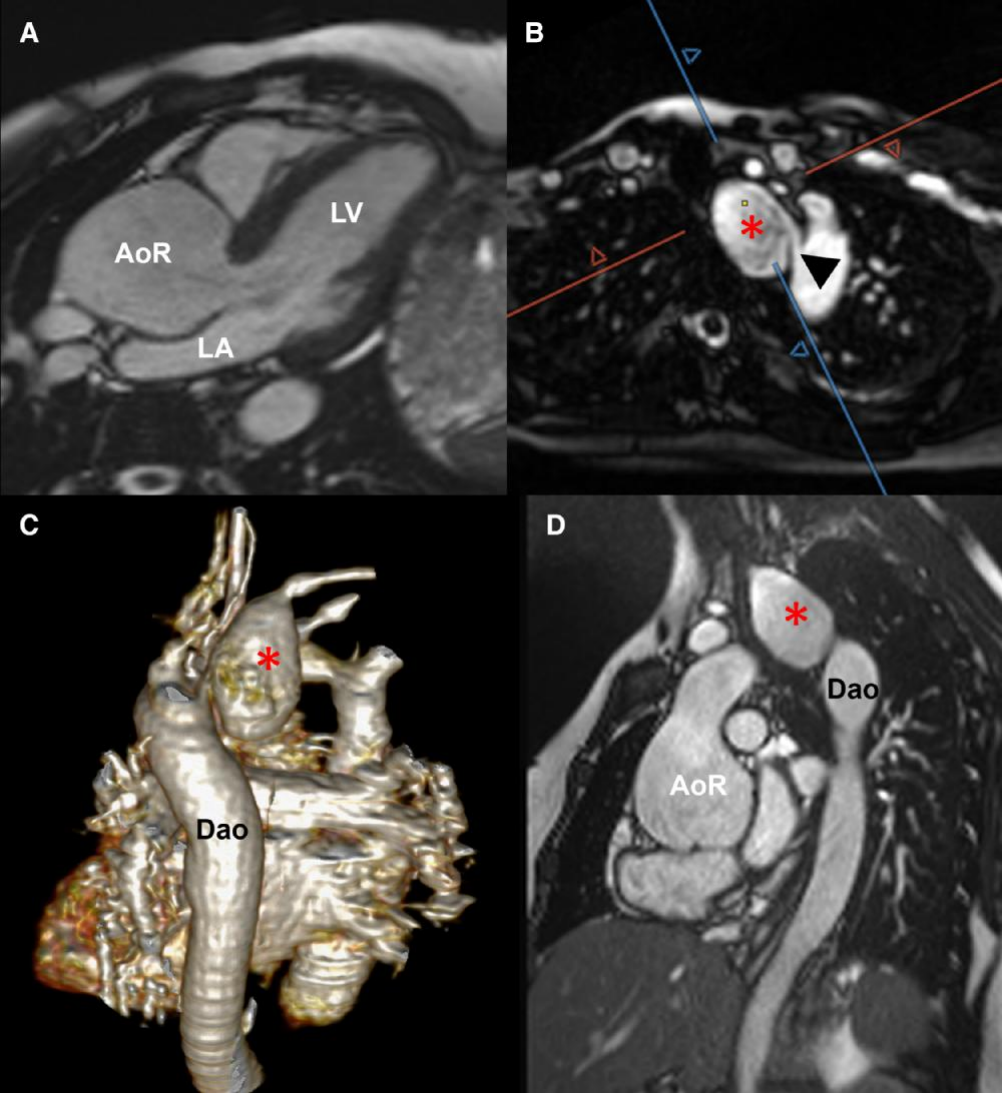
Cardiovascular magnetic resonance imaging without contrast at 21 weeks of gestation. Three-chamber view showing the enlarged aortic root (*A*). Quiescent-interval slice-selective (QISS) (*B*, *C*) and cine MRI (*D*) demonstrating the subclavian artery aneurysm (star) and the origin of the aberrant right subclavian artery (arrowhead). AoR, aortic root; Dao, descending aorta; LA, left atrium; LV, left ventricle; RV, right ventricle.

**Figure 3:**
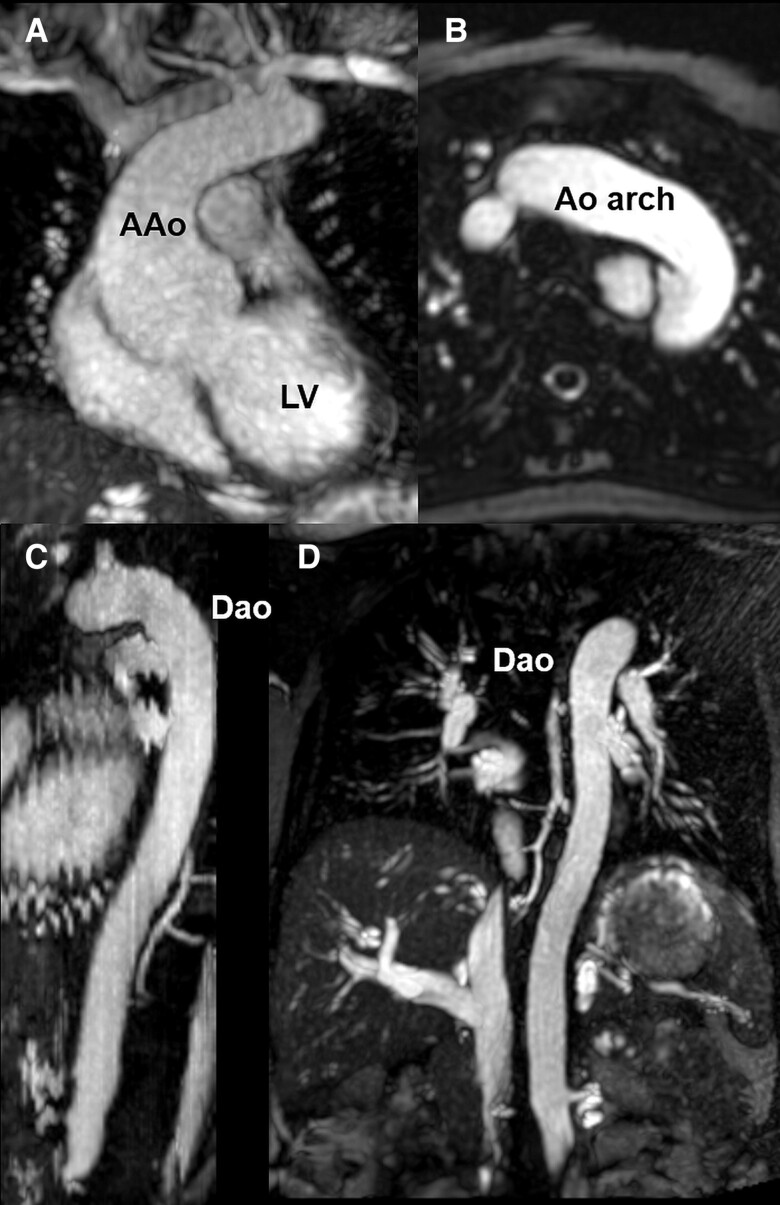
Cardiovascular magnetic resonance images without contrast at 21 weeks of gestation showing the ascending aorta, aortic arch, and descending aorta (*A–D*). AAo, ascending aorta; Dao, descending aorta; LV, left ventricle.

Cardiovascular magnetic resonance performed on the same day confirmed the aortic root dilatation (aortic root cusp-to-cusp 50 × 51 × 55 mm; *Figure 2*) and showed an additional aneurysm of the aberrant right subclavian artery measuring ∼24 × 32 × 48 mm (*Figure 2*, *Movie clips 3*–*6*). The ascending aorta and the aortic arch were not dilated (ascending aorta at right pulmonary artery level 22 × 25 mm, upper ascending aorta 20 × 22 mm, transverse aortic arch 21 × 23 mm), but the proximal descending aorta was mildly enlarged (24 × 26 mm) (*Figures 2* and *3*, *Movie clips 3*–*6*). To avoid contrast application, free-breathing radial quiescent-interval slice-selective magnetic resonance angiography was acquired.^6^ Left ventricular volumes and ejection fraction were in the normal range (left ventricular ejection fraction was 58%).

A multidisciplinary case discussion involving cardiology, cardiovascular surgery, angiology, anaesthesiology, obstetrics, and neonatologists was held to discuss further management considering the risks for mother and foetus involved in the different treatment options (conservative vs. interventional vs. surgical management).

The risk of ascending aortic dissection and rupture was thought to be significant and life-threatening for both the mother and foetus,^1,2,7^ and potentially higher than aortic surgery during pregnancy. Therefore, urgent aortic root replacement during pregnancy was discussed including peri- and intraoperative management for the mother and foetus and the patient consented to the procedure.

It was also felt that the risk for surgical treatment of the aberrant subclavian artery aneurysm during the same procedure would prolong the procedure and increase its risk particularly for the foetus. It was felt that repeat CMR imaging was required during the remainder of the pregnancy to evaluate the potential need for an additional interventional (transcatheter) procedure during or after pregnancy.

Successful valve-sparing aortic root and ascending aorta replacement was performed at 22 + 2 weeks of gestation. For foetal safety, cardiopulmonary bypass temperature was kept near normothermic (35°C) and perioperative foetal echocardiography was performed. Intraoperative inspection of the ascending aorta showed confined dissection of the vessel wall at the level of the pulmonary artery that was not seen on the CMR images. Histopathology of the resected aortic segment confirmed fresh dissection of the aortic wall.

**Figure 4:**
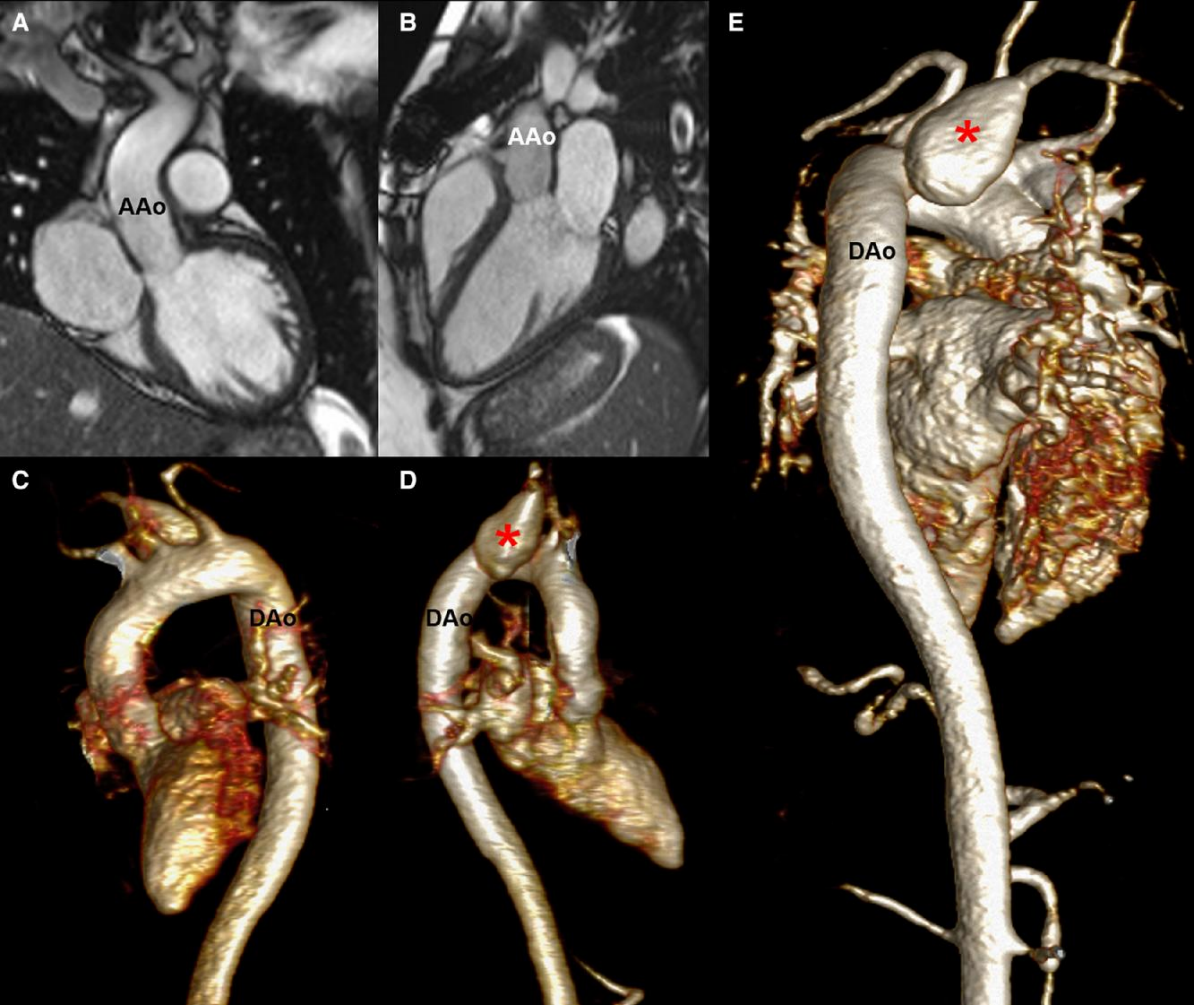
Cardiovascular magnetic resonance images after delivery. Left ventricular outflow tract and three-chamber views demonstrating the replaced ascending aorta (*A*, *B*). Three-dimensional reconstructions (*C–E*) derived from contrast-enhanced magnetic resonance angiography showing the thoracic aorta and the subclavian artery aneurysm (star). AAo, ascending aorta; Dao, descending aorta; LV, left ventricle.

The foetus survived the operation, and post-operative course of the mother was timely. Repeated imaging during pregnancy, peri- and post-partum showed mild aortic regurgitation (regurgitant fraction 7%), and stable dimensions of the aorta (upper ascending aorta 21 × 23 mm, transverse aortic arch 21 × 23 mm, proximal descending aorta 25 × 26 mm) while there was a mild increase in size of the aneurysm of the aberrant right subclavian artery (*Figure 4*).

At 35 weeks of gestation, the patient developed cervical insufficiency and an uncomplicated caesarean section was performed with delivery of a healthy boy.

Post-partum recovery of the mother was unremarkable. The baby, however, was diagnosed with hypertrophic pyloric stenosis and underwent pyloromyotomy before mother and child were discharged in good condition ∼3 weeks after caesarean section. Paediatric cardiology investigations in the child did not show any cardiovascular abnormality.

The subclavian aneurysm was treated successfully by interventional embolization after interdisciplinary team discussions.

Follow-up investigations at 1 and 11 months show good surgical and interventional results, and the patient is in good clinical condition and treated with a beta-blocker to prevent further aortic dilatation.

## Discussion

The main cardiovascular manifestation in patients with Marfan syndrome is dilatation of the aortic root than can lead to type A aortic dissection and aortic rupture with high mortality rates.^8,9^ Dilatation of the descending aorta and type B aortic dissection are less common.^8,10^ Pregnancy in women with Marfan syndrome increases the risk of aortic dissection with high mortality and morbidity for mother and child^1,2,7^ likely related to haemodynamic and hormonal changes. It is therefore recommended that women with Marfan syndrome should be counselled about their risk for aortic dissection prior to pregnancy considering current aortic imaging data.^3,4^ Furthermore, in patients with Marfan syndrome and other genetic arteriopathies, an increased risk for dissection of an aberrant subclavian artery has been reported and this should be considered during pre-pregnancy counselling.^11^

Assessment of the aortic root and ascending aortic diameter in adult Marfan patients is typically performed annually using transthoracic echocardiography. Cardiovascular magnetic resonance or computed tomography (CT) is recommended in addition in patients with reduced visibility of the aortic root as well as the ascending and descending aorta.^4^ Prior to conception, the entire aorta should be visualized using CMR or CT.^4,12^ When thresholds for aortic surgery are approached, more frequent imaging (typically every 6 months) should be performed.^4^ As surgical thresholds are lower in women with Marfan syndrome and desire for pregnancy, many female patients at childbearing age will undergo cardiac follow-up every 6 months.

The pre-pregnancy assessment should also include the aspect if surgery prior to pregnancy is indicated. According to current guidelines, pre-pregnancy aortic surgery is recommended when the ascending aorta has a size of >45 mm.^3,4^ In patients with an ascending aortic size of 40–45 mm, surgery should be considered prior to pregnancy if additional risk factors such as a rapid aortic growth (>0.3 cm/year) or a positive family history for aortic dissection are present. Prophylactic aortic surgery during pregnancy should be considered and discussed in patients with an aortic diameter of >45 mm and a rapid size increase.^3^

Our patient was lost to follow-up, and implications of V were never discussed with her. In a multidisciplinary team approach, we considered the high risk of aortic dissection and the abovementioned guideline recommendations for prophylactic aortic surgery.^3^ Although valve-sparing aortic valve replacement was successful and uneventful for mother and child, mortality rates for cardiac surgery during pregnancy are reported as being high for mother and child and bypass surgery should be avoided.^13,14^

Beside aortic aneurysms, other arterial aneurysms have been reported in Marfan patients.^15^ In our case, the aneurysm was successfully treated by interventional embolization showing that transcatheter treatment can be a good option.

## Conclusion

Pregnancy in patients with Marfan syndrome carries an increased risk for cardiovascular complications, especially aortic dilatation, and dissection. Although aortic surgery was performed successfully during pregnancy in our case, cardiovascular bypass surgery is associated with high mortality. Regular aortic surveillance, pre-conception counselling, and timely aortic surgery prior to pregnancy are strongly advised to minimize the risk for mother and child.

## Lead author biography


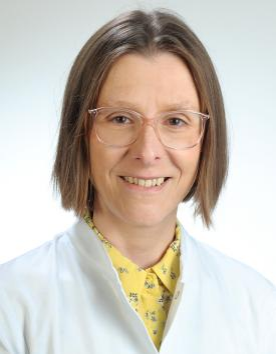


Prof. Inga Voges is a consultant at the Department of Congenital Heart Disease and Pediatric Cardiology at the University Hospital Schleswig-Holstein in Kiel, Germany where she is the lead for the Cardiovascular Magnetic Resonance (CMR) Imaging programme. Her clinical interests are paediatric cardiomyopathies, adult congenital heart disease, and univentricular hearts. Professor Voges is the current chair of the Association for European Paediatric and Congenital Cardiology (AEPC) imaging working group and the chair of the research group of the German Society of Pediatric Cardiology (DGPK). Professor Voges has published several articles with a focus on cardiovascular imaging and congenital heart disease.

## Supplementary Material

ytae411_Supplementary_Data

## Data Availability

The data underlying this article cannot be shared publicly due to the privacy of the individual described in this article.

## Supplementary material

**Consent:** The authors confirm that written consent for submission and publication of this case report including images and associated text has been obtained from the patient in line with COPE guidance.

**Funding:** None.
